# I. Fracture of the Trapezium, or Rupture of One of the Tendons Attached to That Bone. II. Peritonitis1Reported to the Virginia State Veterinary Medical Association, June 18, 1895.

**Published:** 1895-09

**Authors:** 

**Affiliations:** Charlottesville, Va.; Charlottesville, Va.


### I. FRACTURE OF THE TRAPEZIUM, OR RUPTURE OF ONE OF THE TENDONS ATTACHED TO THAT BONE. II. PERITONITIS.[1]

BY DRS. MARSHALL AND DIXON,
CHARLOTTESVILLE, VA.

CASE I. Bay gelding, four years old, previously sound; ridden along country road at a canter, suddenly pulled up dead lame. Found swelling all around the knee; could not put his foot to the ground.

*Diagnosis.* Either fracture of the trapezium, or rupture of one of the tendons attached to that bone.

*Treatment.* Hot fomentations, contined for several days afterwards, followed by cold sprayings. Inflammation having subsided, blistered, which was repeated as soon as possible, colt being in the stable all the time.

Shortly after second blister was turned out and not seen for about three weeks—was then found that the swelling at the trapezium was much larger and appeared to be of an osseous nature. Horse still lame.

CASE II. Chestnut colt, two years old. Altered by a castrator three weeks previously. When called, owner stated that he could not urinate, and was continually straining. Colt appeared very weak and stiff, unable to turn and in great pain; pulse hard and quick; straining a great deal, but saw him make water; on rectal examination found soft, pulpy tumors.

[1] Reported to the Virginia State Veterinary Medical Association, June 18, 1895.

*Diagnosis.* Peritonitis, with other lesions of the rectum, which I was unable to diagnose.

*Treatment.* Advised the owner to kill, as I told him the animal could not live till morning. He, however, declined. Arrived next morning and found that the animal had died during the night.

*Post-mortem.* On opening found the left cord swollen, and part of the epididymis, which was so large that it was mistaken for a small testicle. On following the cord found continuous suppuration and finally came upon a large abscess, which was apparently discharging into the rectum. On opening the sac over a pint of pus escaped. The rectum was then laid bare and and on the mucous membrane were found a number of gelatinous tumors, varying in size from a pea to a hen's egg, chiefly in the lower part. The peritoneum and intestines were much inflamed and a great quantity of effusion escaped in opening the abdominal cavity, Little or no change was observed in the thoracic cavity.

